# Excessive BCAA regulates fat metabolism partially through the modification of m^6^A RNA methylation in weanling piglets

**DOI:** 10.1186/s12986-019-0424-x

**Published:** 2020-01-23

**Authors:** Jinghui Heng, Zhihui Wu, Min Tian, Jiaming Chen, Hanqing Song, Fang Chen, Wutai Guan, Shihai Zhang

**Affiliations:** 1 Guangdong Province Key Laboratory of Animal Nutrition Control, College of Animal Science, South China Agricultural University, Wushan Avenue, Tianhe District, Guangzhou, 510642 China; 2 College of Animal Science and National Engineering Research Center for Breeding Swine Industry, South China Agricultural University, Wushan Avenue, Tianhe District, Guangzhou, 510642 China

**Keywords:** Branched-chain amino acids, Lipid metabolism, m^6^A RNA methylation, Weanling piglets, Low-protein diet

## Abstract

**Background:**

Fat percentage and distribution in pigs are associated with their productive efficiency and meat quality. Dietary branched-chain amino acids (BCAA) regulate fat metabolism in weanling piglets with unknown mechanism. It is reported that N6-methyl-adenosine (m^6^A) is involved in fat metabolism in mice. The current study was designed to investigate the relationship between dietary branched-chain amino acids and fat metabolism through N6-methyl-adenosine (m^6^A) in weanling piglets.

**Methods:**

A total of 18 healthy crossbred weaned piglets (Duroc × Landrace × Large White, 10.45 ± 0.41 kg) were divided into 3 treatments and were fed the low BCAA dose diet (L-BCAA), the normal dose BCAA diet (N-BCAA), or the high dose BCAA (H-BCAA) diet for 3 weeks.

**Results:**

Our results show that compared with the N-BCAA group, the L-BCAA group had higher concentration of serum leptin (*P* < 0.05), while the H-BCAA group had lower concentration of serum adiponectin (*P* < 0.05). Fatty acid synthesis in pigs from the H-BCAA group was lower than those from the N-BCAA group with the down-regulation of lipogenic genes (ACACA, FASN, PPAR-r, SREBP-1c in ventral and dorsal fat, SREBP-1c in liver) and up-regulation of lipolysis genes (HSL, ATGL, CPT-1A, FABP4 in ventral fat, HSL in liver) (*P* < 0.05). Similarly, fatty acid synthesis in pigs from the L-BCAA group was also lower than those from the N-BCAA group with the decrease of lipogenic genes (ACACA in ventral, ACACA and FASN in dorsal fat, ACACA, FASN, SREBP-1c in liver) and the increase of lipolysis genes (ATGL, CPT-1A CD36, FABP4 in ventral fat and HSL, ATGL, CPT-1A in dorsal fat, CPT-1A) (*P* < 0.05). Feeding H-BCAA diet significantly reduced total m^6^A levels in ventral and dorsal fat and liver tissues (*P* < 0.05). The decrease of total m^6^A is associated with down-regulation of METTL3, METTL14 and FTO in dorsal fat and METTL3 and FTO in liver (*P* < 0.05). Decreased m^6^A modification of ACACA and FASN in ventral and dorsal adipose tissues was observed in pig fed with excessive BCAA.

**Conclusion:**

These results suggest that insufficient or excessive BCAA decreased the fat deposition by increasing lipolysis and deceasing lipogenesis in adipose and liver tissues. Dietary excessive BCAA might regulate the process of lipid metabolism partly through the m^6^A RNA methylation.

## Introduction

Feeding pigs with protein-restricted diet is a strategic way to save the cost of protein ingredients and decrease the emission of nitrogen in animal husbandry [1]. However, pigs fed with low-protein diet tend to deposit thicker back fat during growing and fastening periods, which eventually affect carcass characteristics [2, 3]. Decreasing dietary crude protein level by 2–4% from the NRC (1998) recommendation is the most classical method to make protein-restricted diets for pigs. In order to compensate the deficiency of essential amino acids, four crystalline amino acids (L-lysine, DL-methionine, L-threonine, and L-tryptophan) are usually supplemented to the low protein diets [1]. However, there might be a shortage of other functional amino acids which participated in protein synthesis and lipid metabolism in low-protein diets. Branched chain amino acids (BCAAs) are considered as important functional amino acids to improve carcass characteristics of pigs due to its potential to enhance protein synthesis through activating the Sestrin2/mTORC1 (mammalian target of rapamycin complex 1) pathway [4, 5].

Recent studies have shown that BCAAs also play essential roles in energy homeostasis and lipid metabolism [6]. Growing evidence have found that shortage of BCAAs reduces fat deposition in mice. A significant reduction of ventral fat mass is observed in mice consuming a leucine-deficient diet, which is partly caused by the increase of energy expenditure [7]. Furthermore, expression of genes involved in lipogenesis and lipolysis is decreased and increased in these mice, respectively [7]. Similarly, when mice are fed isoleucine- or valine-deficient diets, ventral and whole body fat mass are also decreased along with the up-regulated lipolytic genes [8]. Interestingly, excessive BCAAs also inhibit fat accumulation. Decreased body fat mass is observed in rats fed a high level of leucine diet [9]. Leucine over-supplementation also suppresses fat synthesis in murine adipocytes in vitro [10]. In recent years, studies in growing pigs have demonstrated that different dietary BCAA ratios modulate lipid metabolism in intramuscular fat and adipose tissues, indicating their vital role in fat metabolism in pigs [11, 12].

Lipogenesis and lipolysis are well-regulated pathways and a number of genes have been shown to be involved in these processes. The Fat Mass and Obesity-associated (FTO) is one of the genes associated with body mass index and obesity in children and adult [13]. FTO has been originally reported to contribute to human risk of obesity, mainly through the regulation of food intake [14]. Recently, some studies have demonstrated that FTO knockout in mouse significantly disrupts the progress of adipogenesis, while FTO overexpression in mouse increases adipogenic differentiation in primary adipocytes and mouse embryonic fibroblasts [15]. In addition, an intimate relationship between FTO expression and fat deposition has also been observed in pigs [16]. It is worth mentioning that FTO is also an important enzyme involved in N6-Methyladenosine (m^6^A) RNA modification [17]. m^6^A modification, which promotes mRNA stability, splicing, export, and translation, is considered as the most abundant post-transcriptional modification in eukaryotic mRNA and long noncoding RNA [18]. The biological process of m^6^A is reversible, which is regulated by methyltransferases and demethylases [19]. Methyltransferases, which add methyl groups to m6A, include Wilms’ tumor 1-associating protein (WTAP), methyltransferase like 3 (METTL3), and methyltransferase like 14 (METTL14). These methyl groups from m^6^A can be removed by demethylases [FTO and α-ketoglutarate-dependent dioxygenase alkB homolog 5 (ALKBH5)]. The execution of the function of m^6^A requires the recognition of m^6^A by readers like: YTH domain family (YTHDF) proteins and the heterogeneous nuclear ribonucleoprotein (HNRNP) proteins.

In the current study, we hypothesized that dietary BCAAs might dose-dependently regulate fat metabolism through m^6^A methylation partially through the regulate of the FTO. To test our hypothesis, weanling pigs were assigned to one of the following diets: BCAA-deficient, −normal, or -excessive diets. Changes of rate-limiting enzymes involved in lipolysis, lipogenesis and m^6^A RNA modification in subcutaneous adipose tissue, ventral adipose tissue, and liver were determined in this study. These results provide important information regarding the relationship between dietary BCAAs and carcass characteristics in pigs and its underlying mechanism. Our study also extends our knowledge that m^6^A methylation might act as a novel and critical bridge between dietary nutrients and obesity in human.

## Materials and methods

### Animals and experimental diets

All procedures outlined in this study were conducted under the protocol (SCAU-AEC-2010-0416) approved by the South China Agricultural University Animal Care and Use Committee. In our experiments, a total of 18 healthy crossbred weaned piglets (Duroc × Landrace × Large White, 10.45 ± 0.41 kg) were divided into 3 treatments using a completely randomized design. All piglets were kept in metabolic cages (1.40 × 0.68 × 0.90 m^3^). The formula of three treatments are shown in Table 1. Pigs in the L-BCAA group were fed the low-protein (LP) diet containing 17.05% crude protein (CP) supplemented with L-lysine, L-methionine, L-threonine, and L-serine. N-BCAA and H-BCAA groups were designed based on the L-BCAA group. In N-BCAA group, extra crystal BCAAs were supplemented as recommended by the National Research Council (NRC) (2012) (0.13% L-isoleucine, 0.09% L-leucine, and 0.23% L-valine) to meet the requirement of standardized ileal digestible amino acids (SID AAs). In the H-BCAA diet, BCAAs were supplemented to reach the 150% SID AA requirement according to the NRC (2012). Pigs had free access to water via a nipple drinker throughout the process of this experiment.

**Table 1:** Composition of the experimental diets (As-Fed basis)

Items	Treatments^a^		
	L-BCAA	N-BCAA	H-BCAA
Ingredient (%)			
Corn (7.8%, CP)	69.24	69.38	69.50
Soybean meal (47%, CP)	9.45	9.15	8.55
Concentrated soybean protein (65.2%, CP)	5.00	5.00	5.00
Fish meal (62.5%, CP)	4.00	4.00	4.00
Whey powder (3.8%, CP)	5.00	5.00	5.00
Soybean oil powder	1.50	1.50	1.50
L-lysine HCl	0.66	0.66	0.67
_D,L_-methionine	0.30	0.30	0.31
L-threonine	0.30	0.30	0.32
L-tryptophan	0.08	0.08	0.09
L-leucine	0	0.09	0.82
L-isoleucine	0	0.13	0.51
L-valine	0	0.23	0.70
L-alanine	1.44	1.15	0
Salt	0.30	0.30	0.30
Calcium formate	0.60	0.60	0.60
Dicalcium phosphate	1.13	1.13	1.13
Premix^b^	1.00	1.00	1.00
Calculated Nutrient Content (%)			
Crude protein	17.45	17.45	17.45
Digestive energy (MJ/kg)	14.68	14.70	14.64
Total Ca	0.80	0.80	0.80
Total P	0.66	0.66	0.66
Available P	0.44	0.44	0.44
Lys	1.36	1.35	1.35
Met + Cys	0.75	0.75	0.74
Threonine	0.79	0.79	0.79
Tryptophan	0.22	0.22	0.22
Leucine	1.25	1.35	2.03
Isoleucine	0.55	0.69	1.04
Valine	0.62	0.86	1.29

^a^ *L-BCAA* Low dose BCAA diet, *N-BCAA* Normal dose BCAA diet, *H-BCAA* High dose BCAA diet. ^b^ Provided per kilogram of diet (as-fed basis): VA, 13000 IU; VD_3_, 40 00 IU; VE, 39.4 mg; VB_1_, 6.2 mg; VB_2_, 11.2 mg; VB_6_, 12.2 mg; VB_12_, 4 mg; VK, 4.0 mg; niacin, 45.0 mg; folate, 2.2 mg; pantothenic acid, 25.0 mg; biotin, 0.2 mg; choline chloride, 500.0 mg; Cu, 35.0 mg; Fe, 105.0 mg; Mn, 25.0 mg; Zn, 1600.0 mg; I, 0.3 mg; Se, 0.6 mg; Co, 0.3 mg

### Sample collection

Blood samples were collected from pigs in heparin-free vacutainer tubes at the end of the experiment (fasting state). After blood collection, all samples were centrifuged at 3000 g for 15 min at 4 °C. The serum was obtained and stored at − 80 °C immediately for later analysis. All piglets were sacrificed by electrocution immediately after blood sampling. Ventral, subcutaneous adipose and dorsal subcutaneous adipose were excised from the left side of the carcasses between the sixth and seventh ribs. Liver samples were consistently dissected from right side of whole liver. Adipose and liver tissues were immediately frozen in liquid nitrogen and then stored at − 80 for further analysis.

### Serum biochemical analysis

The concentrations of total cholesterol (TC), high-density lipoprotein-cholesterol (HDL-C), low-density lipoprotein-cholesterol (LDL-C), glucose and triglyceride in serum were measured using corresponding commercial available kits (Nanjing Jiancheng Biochemical Reagent Co., Nanjing, China) through the automatic microplate reader (Thermo Scientific™ Multiskan™ GO, USA). In addition, the concentrations of leptin, insulin and adiponectin in serum samples were measured by the commercial ELISA kits purchased from Cusabio Biotech Co., Ltd. (Wuhan, China).

### RNA extraction and purification

Cytoplasmic RNA was isolated from ventral subcutaneous adipose, dorsal subcutaneous adipose, and liver of piglets using the cytoplasmic & nuclear RNA purification kit according to the manufacturer’s protocol (NORGEN, Canada, North American). The concentration of extracted RNA was measured by Nano Drop spectrophotometer (Nano Drop Technologies, Wilmington, DE, USA). We also checked the integrity of mRNA by 1% agarose gel electrophoresis. The mRNA was reverse-transcribed to complementary DNA (cDNA) with the PrimeScript 1st Strand cDNA Synthesis Kit (Takara, Dalian, Liaoning, China) according the manufacturer’s protocol. After that, the synthesized cDNA was stored at − 20 °C for further real-time PCR analysis.

### Gene expression using RT-PCR

The real-time polymerase chain reaction (RT-PCR) was conducted using an ABI Prism 7500 sequence detection system (Applied Biosystems, Carlsbad, CA). This procedure was performed in a 20 μL reaction volume, containing 10 μL SYBR Green PCR Master Mix (Takara, Dalian, Liaoning, China), 2 μL cDNA, 0.8 μL of each PCR primer (10 μM), 0.4 μL ROX (Dalian, Liaoning, China), and 6 μL dd H_2_O. The cycling conditions for polymerase chain reaction were as follows: (1) incubation for 5 min at 94 °C, followed by (2) 40 repeated cycles of 94 °C for 30 s, (3) annealing at 60 °C for 30 s and extension at 72 °C for 20 s. The mRNA expression level of the target genes was calculated through the 2^−ΔΔCt^ method. Gene-specific primer sequences used for the RT-PCR detection are listed in Table 2, which were synthesized by Sangon Biotech Co. Ltd. (Shanghai, China).

**Table 2:** Primer sequences used in quantitative real-time PCR assay

Primers	Accession No.	Sequences (5′ → 3′)	Product size (bp)
PPAR-γ	NM_214379.1	F-AACATTTCACAAGAGGTGACCA	213
		R-GATCTCGTGGACGCCATACT	
SREBP-1c	NM_214157.1	F-AGCGGACGGCTCACAATG	121
		R-CGCAAGACGGCGGATTTA	
ACACA	NM_001114269.1	F-ACATCCCCACGCTAAACA	186
		R-AGCCCATCACTTCATCAAAG	
FASN	NM_001099930.1	F-GCTTGTCCTGGGAAGAGTGTA	115
		R-AGGAACTCGGACATAGCGG	
CD36	NM_001044622.1	F-ACCCTGAGACCCACACAGTC	119
		R-TACAGCTGCCACAGCCAGAT	
FABP4	NM_001002817.1	F-TGAAAGGTGTCACGGCTAC	102
		R-TCGGGACAATACATCCAACAGAG	
CPT-1A	XM_021091195.1	F-ATTACGACGGCAGGCTGTTGAAG	133
		R- AATAGGCTTGGCGACACTTGGC	
HSL	NM_214315.3	F-GCCCGAGACGAGATTAGC	143
		R-ATGAAGGGATTCTTGACGATG	
ATGL	no. EU373817	F-CGCCAGCATCATCGAGGTGTC	94
		R-GCAGCCACGGATGGTCTTCAC	
DGAT1	NM_214051.1	F-GCTTCAGCCTTCTTCCACGAGTAC	163
		R-CGATGATGAGCGACAGCCACAC	
METTL3	XM_003128580.5	F-CCAGCACAGCTTCAGCAGTTCC	129
		R-TGGAGATGGCAAGACGGATGGAG	
METTL14	XM_003129231.6	F-GAGGAAGAGGTGGAACTTCTGCTG	96
		R-CTCCTCCACGGCCTCCTCTG	
WTAP	NM_001244241.1	F-GCGGGAATAAGGCCTCCAAC	136
		R-TGTGAGTGGCGTGTGAGAGA	
FTO	NM_001112692.1	F-GCAGAGCCGCCTACAACCTAAC	142
		R-ACCGCTGACCTGTCCACCAG	
ALKBH5	XM_021067995.1	F-GCAAGGTGAAGAGCGGCATCC	128
		R-GTCCACCGTGTGCTCGTTGTAC	
YTHDF1	XM_021078235.1	F-GTCTACCTGCTCTTCAGCGTCAAC	139
		R-AGATCCACTTCACGTCGAACTTGC	
YTHDF2	XM_005665152.3	F-CCACCTCCACCACAGCCTACTC	140
		R-CCAGCCTGAGACTGTCCTACTCC	
YTHDF3	XM_021089300.1	F-ATTGAGCAAGGCATGACTGGACTG	140
		R-GGCGCTGCACTGCTAACTGG	
β-actin	397,563	F-TGCGGGACATCAAGGAGAAG	176
		R-AGTTGAAGGTGGTCTCGTGG	

### Western blotting analysis

Western blotting analysis was conducted to detect relative protein levels for METTL3, METTL14, FTO, ALKBH5 and YTHDF2 which are associated with RNA methylation. First, all proteins samples were homogenized on ice with RIPA Lysis Buffer (Beyotime, Shanghai, China), which contains 150 mM NaCl, 50 mM Tris-HCl (pH = 7.4), 1% sodium deoxycholate, 1% Triton X-100, 0.1% SDS and some specific protease inhibitors. A BCA Protein Assay Kit (Beyotime, Shanghai, China) was used to detected total protein concentrations of each sample. Subsequently, a total of 30 μg of protein from each tissue was separated by electrophoresis on SDS-PAGE gels. After transferring to nitrocellulose membranes, all strips were blocked in 5% skimmed milk for 1 h at room temperature. Next, blots were incubated with the following primary antibodies at 4 °C overnight with gently shaking: METTL3, ALKBH5 and MTTL14 (1:1000; Abcam, Cambridge, MA, USA), FTO and β-actin (1:1000; Santa Cruz Biotechnology, USA), YTHDF2 (1:1000, Millipore, Bedford, MA, USA). Subsequently, the membranes were incubated with the corresponding secondary antibody (1: 5000 dilution) (Thermo Fisher Scientific, Rockford, IL, USA) for 1 h at room temperature. Finally, proteins on membrane were developed by the ECL Plus chemiluminescence detection kit (Applygen Technologies, Beijing, China) and analyzed by Image Processing Software (Image Pro Plus 6.0) (Rockville, MD, USA).

### Measurement of m^6^A content

The total content of m^6^A in adipose and liver tissues were determined by an EpiQuik™ m^6^A RNA methylation quantification kit (Epigentek Group Inc. USA) following the recommended procedures. The total content of m^6^A was calculated using the formula: m^6^A % = {[(OD _Sample_ –OD _NC_)/S] ÷ [(OD _PC_ – OD _NC_)]} × 100%, and S, PC and NC represents the total amount of inputting RNA, positive and negative controls, respectively.

### m^6^A immunoprecipitation QPCR of target genes

In order to measure the m^6^A modification of genes involved in lipogenesis and lipolysis, m^6^A immunoprecipitation was performed. Briefly, m^6^A antibody (Abcam, Cambridge, MA, USA) and Normal rabbit IgG (Abclonal, Wuhan China) were conjugated to protein A/G mixed magnetic beads (Bio-rad Laboratories, Hercules, CA, USA) at 4 °C overnight. RNA was fragmented and incubated with the magnetic beads in immunoprecipitation buffer (10 mM Tris, 0.5% NP-40, and 150 mM NaCl) supplemented with RNase inhibitor at 4 °C for 3 h. After washing twice with IP buffer, mRNA was eluted from the beads by elution buffer (5 mM Tris·HCl, 1 mM EDTA, and 0.05% SDS). After extraction and precipitation, the input RNA and eluted RNA were reverse transcribed, and its abundance was determined by RT-PCR.

### Statistical analysis

Data of this experiments were analyzed with one-way ANOVA according to the procedure GLM of SAS (SAS Institute, Cary, NC, USA). Differences among treatment means were determined using Student-Newman-Keuls (SNK) multiple range test. All results are presented as means with their standard error of mean (SEM). *P* value less than 0.05 was considered as significant and effects were considered as tendency when 0.05 ≤ *P* ≤ 0.10.

## Result

### Lipid-related hormone levels in serum

Plasma hormone levels of insulin, leptin and adiponectin are shown in Table 3. Compared with pigs fed the N-BCAA diet, plasma leptin levels were significantly increased in pigs from the L-BCAA groups (*P* < 0.05). In comparison with the N-BCAA diet, plasma adiponectin levels did not change in the L-BCAA group, but significantly increased in the H-BCAA group (*P* < 0.05). There was no difference in plasma insulin level among different groups (*P* > 0.05).

**Table 3:** Lipid-related hormone levels in serum

Projects	Diets			*P*-value
	L-BCAA	N-BCAA	H-BCAA	
Insulin	5.03 ± 0.28	5.13 ± 0.55	6.07 ± 0.60	0.31
Leptin	1.02 ± 0.09^a^	0.44 ± 0.05^b^	0.32 ± 0.02^b^	< 0.01
Adiponectin	15.70 ± 1.23^a^	16.26 ± 3.67^a^	6.63 ± 0.23^b^	0.02

Serum hormone parameters. Diet treatments: *L-BCAA* Low dose BCAA diet, *N-BCAA* Normal dose BCAA diet, *H-BCAA* High dose BCAA diet. Values are means of four pens of four pigs per diet. ^a-b^ Mean values within a line with different superscript letters were significantly different (*p* < 0.05)

### Serum biochemical parameters

Serum biochemical parameters are shown in Table 4. Compared with the L-BCAA group, serum concentrations of T-CHO were lower in the H-BCAA group (*P* < 0.05). Both serum concentrations of HDL-C and LDL-C were obviously lower in pigs fed the L-BCAA diet (*P* < 0.05) compared with those fed the N-BCAA diet. However, no difference was found in plasma TG and glucose levels among different BCAA dose groups (*P* > 0.05).

**Table 4:** Lipid-related biochemical parameters in serum

Projects	Diets			*P*-value
	L-BCAA	N-BCAA	H-BCAA	
TCHO	8.53 ± 0.28^a^	7.63 ± 0.75^ab^	6.47 ± 0.44^b^	0.06
HDL-C	1.48 ± 0.11^b^	2.29 ± 0.20^a^	1.60 ± 0.25^b^	0.04
LDL-C	1.30 ± 0.12^b^	2.72 ± 0.71^a^	0.91 ± 0.25^b^	0.03
Glucose	5.85 ± 0.15	4.87 ± 0.86	3.54 ± 0.98	0.15
TG	0.87 ± 0.05	0.76 ± 0.08	0.86 ± 0.07	0.42

Serum biochemical parameters. Diet treatments: *L-BCAA* Low dose BCAA diet, *N-BCAA* Normal dose BCAA diet, *H-BCAA* High dose BCAA diet, *TCHO* Total cholesterol, *HDL-C* High-density lipoprotein-cholesterol, *LDL-C* Low density lipoprotein-cholesterol, *TG* Triglyceride. Values are means of four pens of four pigs per diet. ^a-b^ Mean values within a line with different superscript letters were significantly different (*p* < 0.05)

### Expression of genes involved in fat metabolism in adipose and liver tissues

**Fig. 1:**
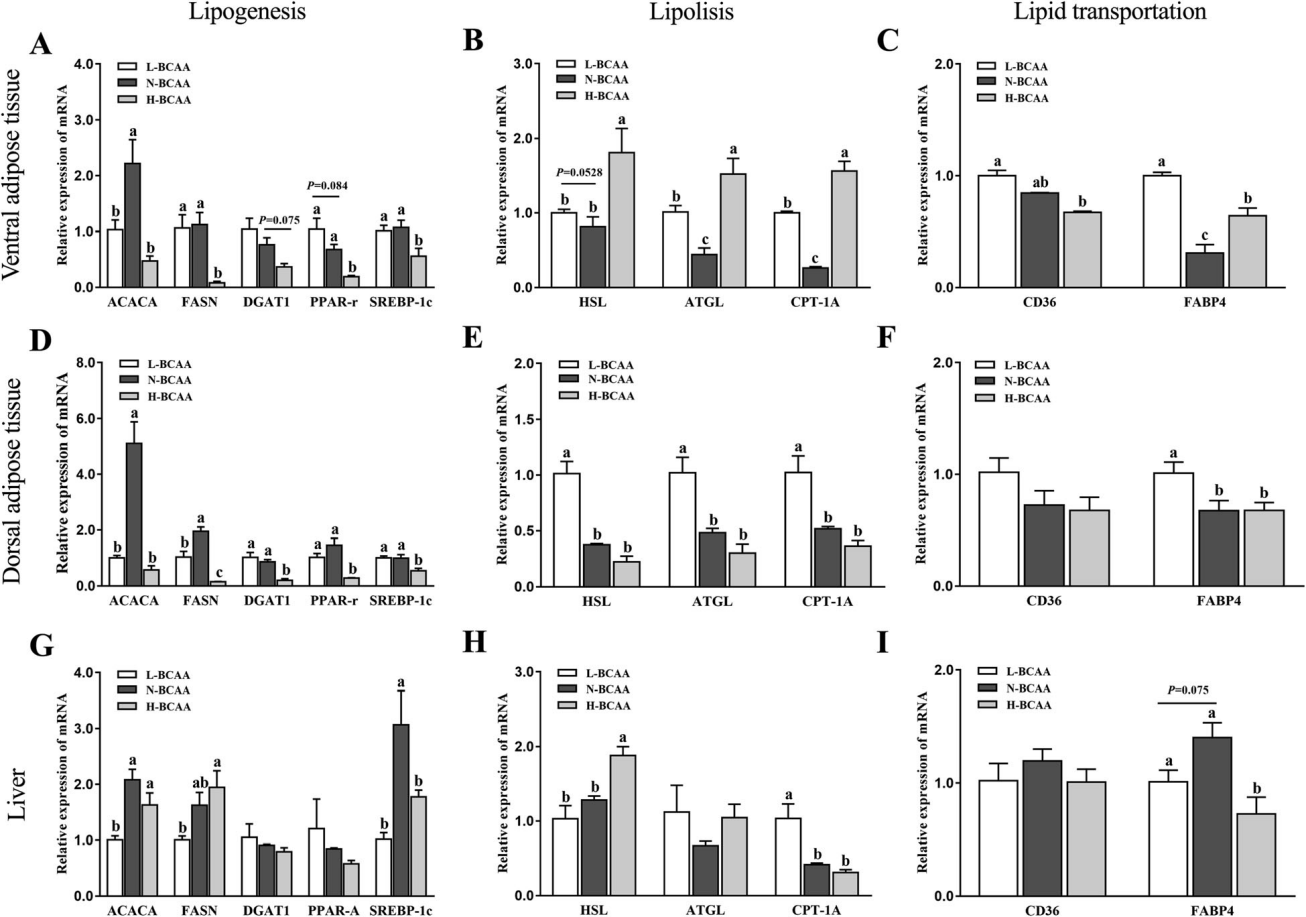
Effect of dietary BCAA level on the expression of genes involved in fat metabolism in ventral subcutaneous adipose (**a**, **b**, **c**), dorsal subcutaneous adipose (**d**, **e**, **f**) and liver (**g**, **h**, **i**). Data are shown as mean ± SEM (*n* = 4). Different letters indicate a significant difference among different treatments (*P* < 0.05)

Figure 1 shows the expression level of genes associated with lipid metabolism in adipose and liver tissues. In ventral subcutaneous adipose tissue, the mRNA level of ACACA in L-BCAA treatment was lower than that in the N-BCAA treatment (*P* < 0.05) (Fig. 1a). Also, the mRNA expression of ACACA, FASN, PPAR-γ and SREBP-1c were lower in the H-BCAA group when compared with the N-BCAA group (*P* < 0.05) (Fig. 1a). Furthermore, the mRNA expression of ATGL and CPT-1A involved in fat hydrolysis (*P* < 0.05) (Fig. 1b) as well as FABP4 and CD36 (*P* < 0.05) (Fig. 1c) involved in fatty acid transport were significantly increased in piglets fed the L-BCAA diet compared with those fed the N-BCAA diet. Similarly, the mRNA expression of HSL, ATGL, CPT-1A (*P* < 0.05) (Fig. 1b) and FABP4 (*P* < 0.05) (Fig. 1c) were higher in the H-BCAA group than the N-BCAA group. In dorsal subcutaneous adipose tissue, the L-BCAA group significantly inhibited ACACA and FASN mRNA expression level (*P* < 0.05) (Fig. 1d) when compared with the N-BCAA group. Also, the H-BCAA group have lower mRNA expression of ACACA, FASN, DGAT1, PPAR-r and SREBP1c than the N-BCAA group (*P* < 0.05) (Fig. 1d). In addition, when compared with the N-BCAA group, pigs from the L-BCAA group have significantly higher mRNA expression of HSL, ATGL, CPT-1A (*P* < 0.05) (Fig. 1e) and FABP4 (*P* < 0.05) (Fig. 1f). In liver tissue samples, pigs fed the L-BCAA diet had lower expression of lipogenic genes (ACACA and SREBP-1c) (*P* < 0.05) (Fig. 1g) and higher expression of lipolysis genes (CPT-1A) (*P* < 0.05) (Fig. 1h) than those fed the N-BCAA diet. While pigs fed with H-BCAA diet have lower level of SREBP-1c (*P* < 0.05) (Fig. 1g) and FABP4 (*P* < 0.05) (Fig. 1i).

### Total methylation levels in adipose and liver tissues

**Fig. 2:**
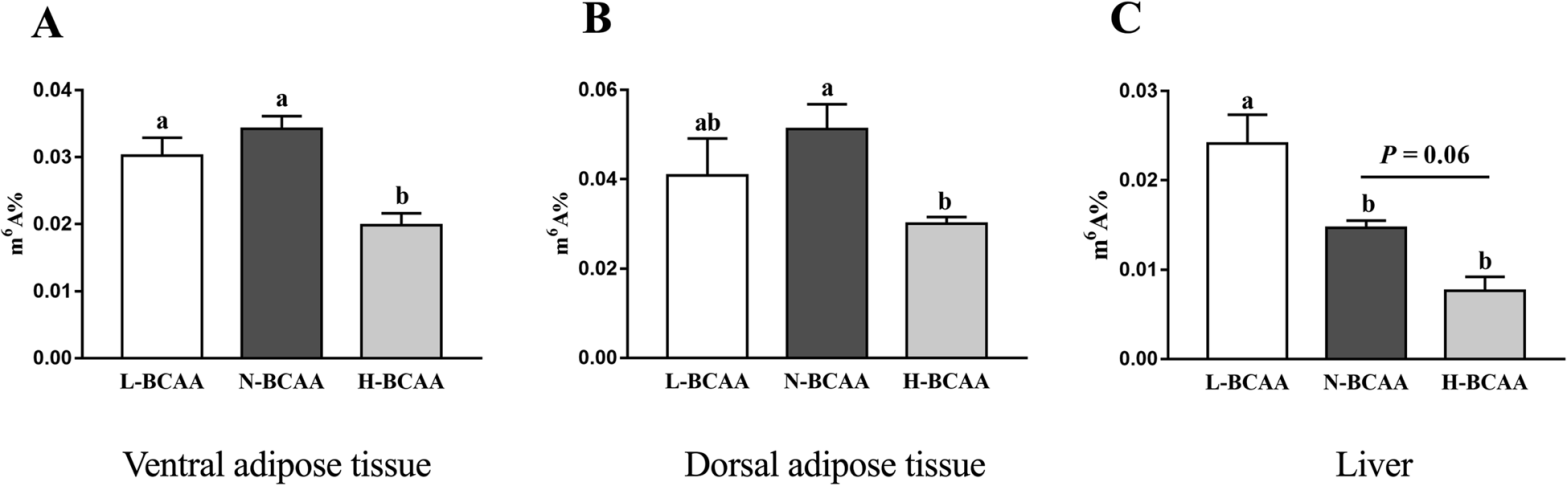
Effect of dietary BCAA level on m6A RNA methylation in ventral subcutaneous adipose (**a**), dorsal subcutaneous adipose (**b**) and liver (**c**). Data are shown as mean ± SEM (*n* = 6). Different letters indicate a significant difference among different treatments (*P* < 0.05)

As shown in (Fig. 2), in ventral subcutaneous adipose tissue and dorsal subcutaneous adipose tissue, compared with the N-BCAA group, total amount of m^6^A was significantly reduced in the H-BCAA group (*P* < 0.05) (Fig. 2a and b), In liver, compared with the N-BCAA group, total amount of m^6^A were higher in the L-BCAA group (*P* < 0.05) (Fig. 2c).

### mRNA expression of m^6^A related enzymes

**Fig. 3:**
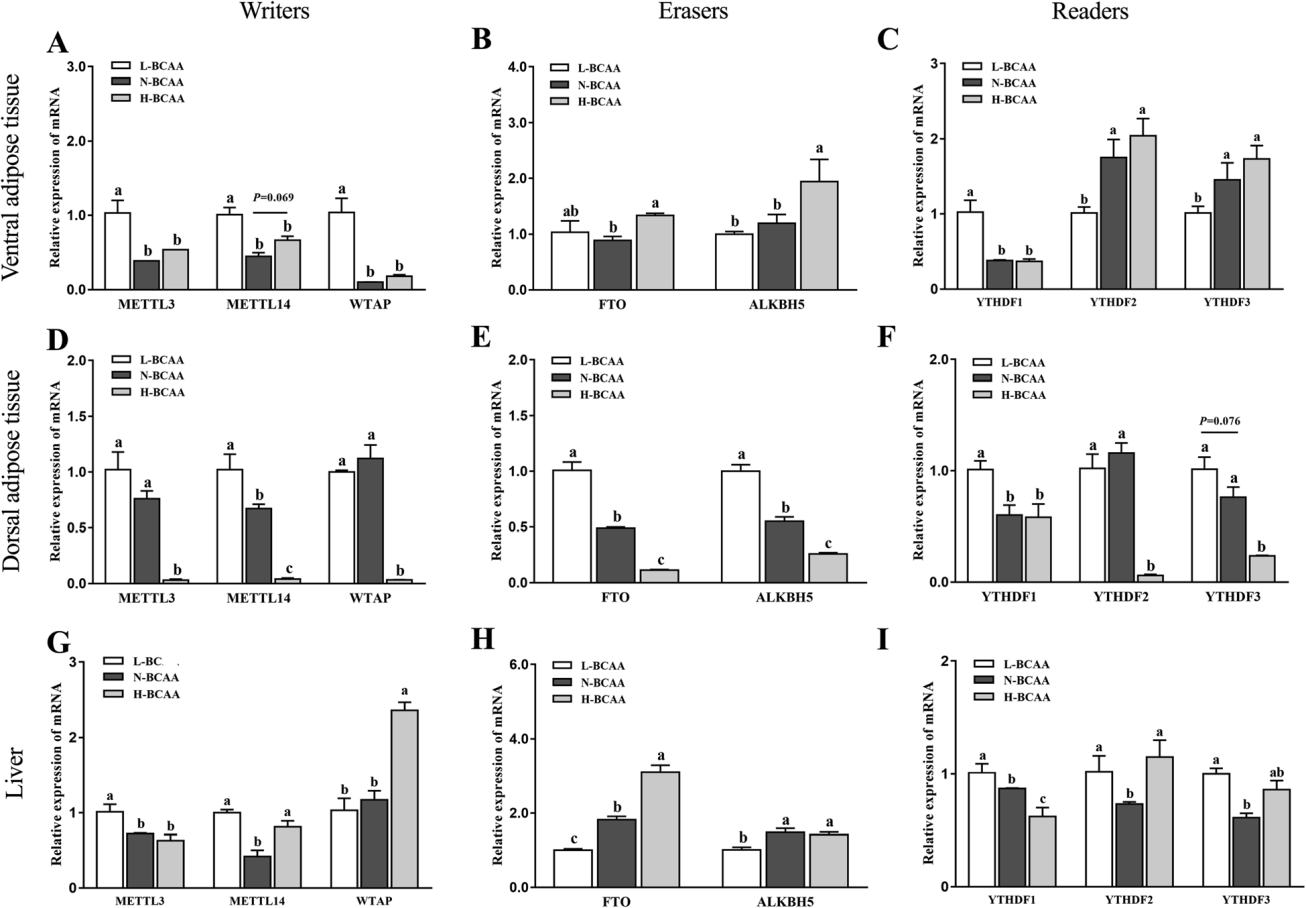
Effect of dietary BCAA level on the expression of genes related to m^6^A RNA methylation in ventral subcutaneous adipose (**a**, **b**, **c**), dorsal subcutaneous adipose (**d**, **e**, **f**) and liver (**g**, **h**, **i**). Data are shown as mean ± SEM (*n* = 6). Different letters indicate a significant difference among different treatments (*P* < 0.05)

The expression of m6A related enzymes are listed in (Fig. 3). In ventral subcutaneous adipose tissue, the mRNA levels of FTO and ALKBH5 (*P* < 0.05) (Fig. 3a, b, and c) were significantly increased in the H-BCAA group when compared with the N-BCAA group. Compared with the N-BCAA group, L-BCAA group increased mRNA expression of METTL3, MELLT14, WTAP as well as YTHDF1 and decreased mRNA expression of YTHDF2 and YTHDF3 in the ventral subcutaneous adipose tissue (Fig. 3a, b, and c). In dorsal subcutaneous adipose, piglets fed with the H-BCAA diet had lower mRNA expression of METTL3, METTL14, WTAP, FTO, ALKBH5, YTHDF2 and YTHDF3 when compared with pigs fed with the N-BCAA diet (Fig. 3d, e and g). However, piglets fed with the L-BCAA diet have higher mRNA levels of METTL14, FTO, ALKBH5, and YTHDF1 than the piglets fed with the N-BCAA diet (Fig. 3d, e and g). In liver tissue, piglets fed the H-BCAA diet showed higher mRNA levels for METTL14, WTAP, FTO, YTHDF2 (*P* < 0.05) (Fig. 3g) compared to those fed with the N-BCAA diet. In addition, compared with the N-BCAA group, pigs fed the L-BCAA group significantly decreased FTO and ALKBH5 expression, but increased METTLE3, METLE14, YTH family protein 1, 2, and 3 expression in liver tissue.

### Protein abundance of m^6^A related enzymes

**Fig. 4:**
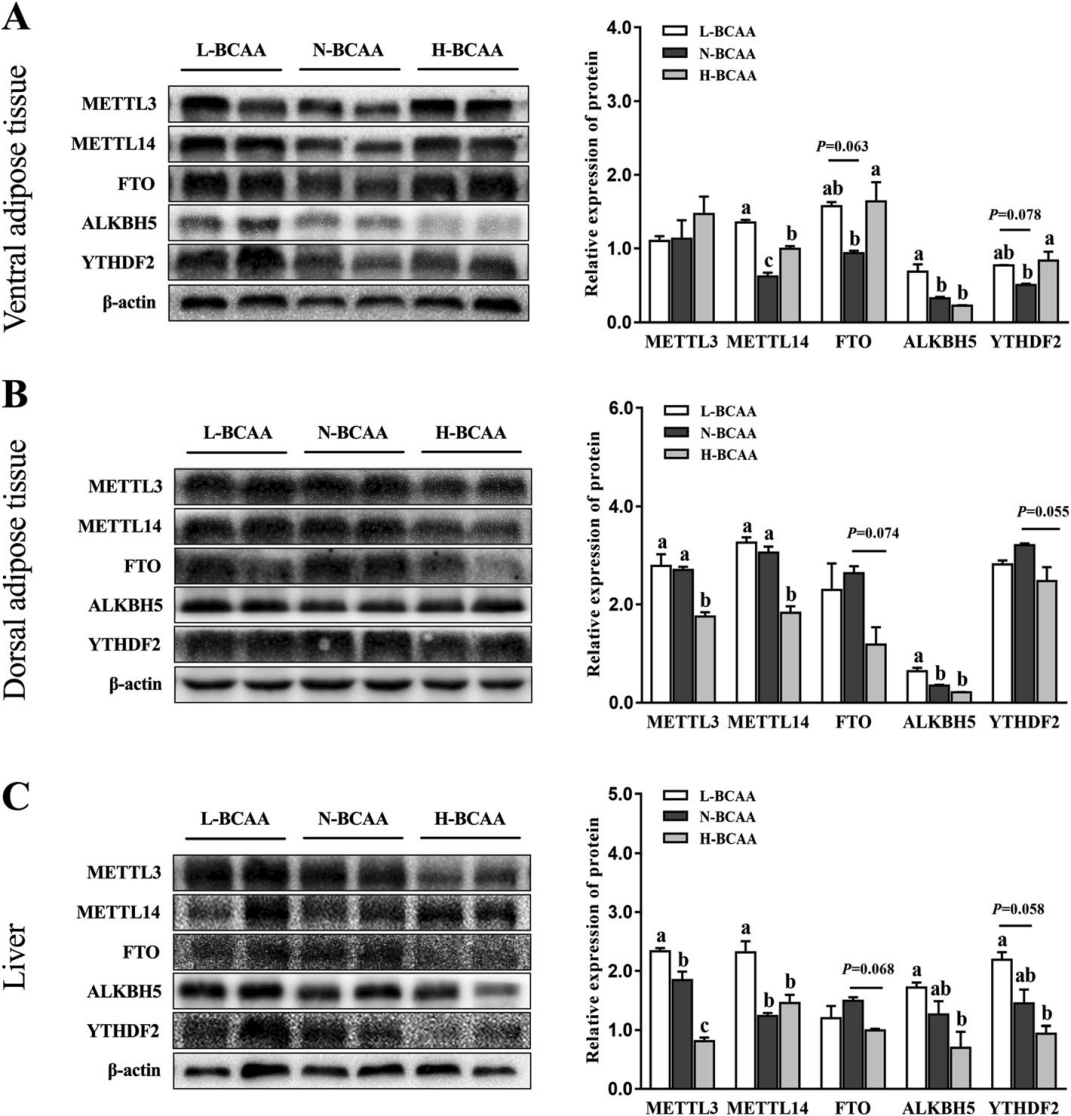
Effect of dietary BCAA level on METTL3, METTL14, FTO, ALKBH5 and YTHDF2 protein level in ventral subcutaneous adipose (**a**), dorsal subcutaneous adipose (**b**) and liver (**c**). Data are shown as mean ± SEM (*n* = 3). Different letters indicate a significant difference among different treatments (*P* < 0.05)

The proteins abundance of METTL3, METTL14, FTO, ALKBH5 and YTHDF2 in adipose and liver tissues are presented in (Fig. 4). In ventral subcutaneous adipose tissue, protein levels of METTL14 and ALKBH5 were higher in the L-BCAA group than the N-BCAA group. The protein levels of MELLT14, FTO and YTHDF2 were higher in the H-BCAA group than the N-BCAA group. (*P* < 0.05) (Fig. 4a). In dorsal subcutaneous fat tissue, the protein amounts of METTL3 and METTL14 were obviously decreased (*P* < 0.05) (Fig. 4b) in the H-BCAA group, when compared with the N-BCAA group. Notably, the protein abundance of ALKBH5 in the L-BCAA group was higher than that in the N-BCAA group (*P* < 0.05) (Fig. 4b). In liver tissue, compared with the N-BCAA group, pigs fed the L-BCAA diet had higher protein levels of METTL3 and METTL14 (*P* < 0.05) (Fig. 4c), while pigs fed the H-BCAA diet inhibited the protein abundance of METTL3 (*P* < 0.05) (Fig. 4c).

### m^6^A modification on lipid metabolism associated genes in adipose tissue

**Fig. 5:**
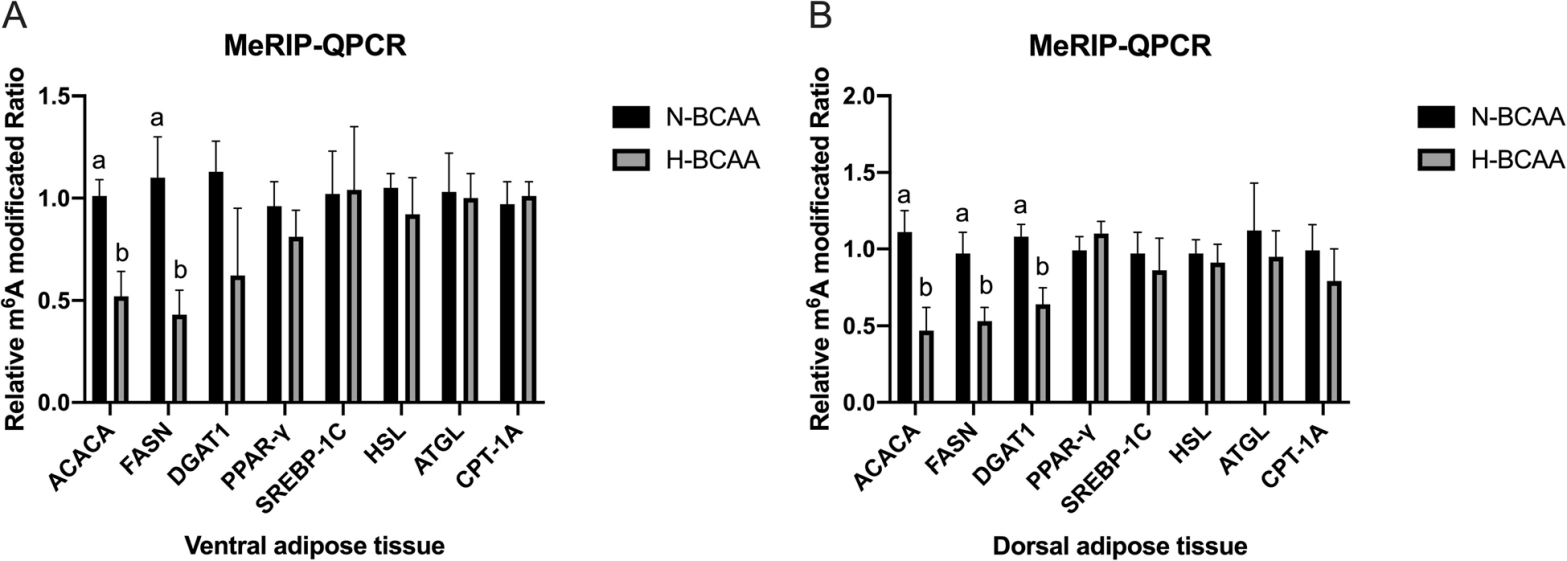
Effect of excessive BCAA on m^6^A modification of genes involved in lipogenesis and lipolysis in ventral (**a**) and dorsal adipose tissues (**b**). Data are shown as mean ± SEM (*n* = 3). Different letters indicate a significant difference among different treatments (*P* < 0.05)

The effect of excessive BCAA on m^6^A modification of genes involved in lipogenesis and lipolysis in adipose tissues is presented in Fig. 5. Compared with the N-BCAA group, the H-BCAA group significantly decreased the m^6^A modification of ACACA and FASN in ventral adipose tissue (*P* < 0.05) and ACACA, FASN and DGAT1 in dorsal adipose tissue (*P* < 0.05).

## Discussion

BCAAs are traditionally considered as functional amino acids that regulate protein synthesis via mTORC1 [6]. Recently, BCAAs have been reported to participate in fatty acid metabolism [7]. However, the underlying mechanism is still unclear. In the current research, the BCAA dose-dependent experiment was conducted to explore the effect of dietary BCAA deficient or excess on fat metabolism in fat and liver and its related mechanism.

Lipid related hormones are affected by different dietary BCAA levels in our study. Adipose tissue is not only an organ for energy storage, but also an endocrine organ which secretes different biologically active molecules into the circulation. Leptin and adiponectin are the two most important fat-derived hormones that participate in energy metabolism [20, 21]. Plasma leptin and adiponectin concentrations are positively and negatively correlated with body fat storage, respectively [22, 23]. In our study, piglets fed the BCAA deficient diet significantly increased the level of leptin, while a lower level of adiponectin was observed from the piglets fed the BCAA excessive diet. In consistent with our results, Li, Wei [24] also found plasma leptin concentration decreases in the BCAA-deficient group. Although the relationship between plasma adiponectin and dietary BCAA level is unclear, an intimate relationship was found between them in the current study. Duan, Li [12] reported that plasma adiponectin concentrations change with different dietary BCAA ratios. Furthermore, leucine metabolite β-hydroxy-β-methylbutyrate regulates fat metabolism partly through adiponectin. Together, these results indicate that unbalanced BCAA might disrupt the whole-body energy metabolism through the modification of leptin and adiponectin secretion. In addition, HDL-C and LDL-C that participated in fat metabolism are also regulated by different dietary BCAA concentrations in our study.

To further study the effect of dietary BCAA on fat metabolism in piglets, genes related to lipolysis and lipogenesis were analyzed in our study. Lipogenesis consists of a series of enzymes, such as ACACA, FASN, DGAT1, SREBPs and PPARs. First of all, acetyl CoA (converted from glucose) is carboxylated into malonyl-CoA by acetyl-CoA carboxylase (ACACA) [25]. Subsequently, fatty acids are synthesized by fatty acid synthase (FASN) with acetyl-CoA, malonyl-CoA and NADPH [26]. Finally, three free fatty acids and one glycerol are combined into a triglyceride under diacylglycerol acyltransferase (DAGT) catalysis as the final step of lipogenesis [27]. This lipogenesis process is regulated by several important regulators. For instance, sterol regulatory element–binding proteins (SREBPs), a membrane-bound transcription factor, can strongly increase the expression of genes involved in synthesis and uptake of fatty acids [28]. Furthermore, nuclear receptor peroxisome proliferator-activated receptors (PPARs) also enhance fatty acid synthesis by increasing the expression of fatty acid transport protein and acyl-CoA synthetase [29]. Lipolysis is also a well-regulated process, which includes fatty acid mobilization and oxidation. Free fatty acids are released from triacylglycerol by hormone-sensitive lipase (HSL) and adipose triglyceride lipase (ATGL) [30, 31] and are then transported into the cell by FABP4 and FAT/CD36 [32–34]. After the production of acyl CoA from free fatty acid, the step-limiting enzyme carnitine Palmitoyl Transferase-1 (CPT1) transports acyl-CoA into mitochondria for β-oxidation [35].

In this study, decreased lipogenesis and enhanced lipolysis gene expression was observed in piglets fed both BCAA deficient and excess diets. The inhibition of fat synthesis in adipose tissue have also been reported by many other groups using animal models or cell lines in BCAA deficient situation [7, 8, 36, 37] or excessive situation [9, 10, 38, 39]. Similar effects are also observed in the liver. In broiler chicken, low BCAA decreased the lipogenesis and increased lipolysis in the liver partly through AMPK-mTOR-FoxO1 pathway [40]. In addition, a research conducted in mice reveals that supplementation of BCAA decreases the hepatic TG and lipid droplet size and inhibits the expression of lipogenesis gene [41]. Three interesting observations in the current study are worth noting. First of all, more lipid metabolism genes were changed in piglets fed BCAA excess diet than deficient diet. For example, genes participated in lipogenesis (ACACA, FASN, DAGT1, PPAR-γ and SREBP-1c) were significantly decreased both in fat tissues when piglets fed BCAA excess diet, while only ACACA was down-regulated when piglets fed BCAA deficient diet. In addition, the degree of the decrease in gene expression was greater in the BCAA excess group than in the BCAA deficient group. Finally, the effects of BCAA on gene expression were tissue dependent. For instance, although both ventral subcutaneous adipose tissue and dorsal subcutaneous adipose tissue are belonging to fat, pigs fed BCAA deficient diet only increased the lipolysis genes (HSL, ATGL, and CPT-1A) in ventral subcutaneous adipose tissue, but not in dorsal subcutaneous adipose tissue. However, the underlying mechanism of this phenomenon is still unknown and needs further research.

To test the possible role of m^6^A RNA modification in this experiment, the total m^6^A methylation level and critical enzymes involved in m^6^A RNA modification were tested in our study. We found that only high level of BCAA regulated the m^6^A RNA methylation in adipose tissues. Interestingly, our study also observed that BCAA dose-dependently decreased the m^6^A RNA methylation in the liver. FTO, a m^6^A demethylase, has been found to have positive correlation with fat deposition [42]. The expression of FTO in subcutaneous fat and liver were decreased in the H-BCAA group, which is consistent with the inhibition of the fatty acid deposition in this research. Intriguingly, we observed that down-regulated FTO is accompanied with the decrease of the total m^6^A methylation levels in subcutaneous fat and liver in piglets. The seemingly contradictory results may imply that in addition to FTO, other methyltransferases and demethylases might be involved in the process and need further investigation. We further measured other enzymes and found methyltransferases METTL3 (liver and dorsal subcutaneous adipose tissue), MELLT14 (dorsal subcutaneous adipose tissue) also significantly decreased in the H-BCAA group. The decreased expression of methyltransferases may explain the decreased level of m^6^A methylation. Interestingly, in this study, although excess BCAA diet inhibited lipogenesis in ventral subcutaneous adipose tissue, the expression of FTO was unexpected up-regulated for unknown reason. Collectively, the present study indicates: 1. high dose BCAA treatment decrease the level of m^6^A RNA methylation in adipose tissue and liver, 2. The effect of BCAA on demethylase and methyltransferase is tissue specific. m^6^A RNA methylation is commonly considered to be regulated by dietary methyl donors like: methionine, choline and betaine [43]. To our knowledge, our study was first to report that BCAAs affect m^6^A RNA methylation which might further regulate fat metabolism. Furthermore, decreased m^6^A modification of ACACA and FASN in ventral and dorsal adipose tissues was observed in pig fed with excessive BCAA. Future studies are needed to clarify the changes of m^6^A methylation level of other lipid metabolism associated genes which might participated in fat metabolism.

## Conclusion

BCAA deficient or excess diet decrease the fat deposition by increasing lipolysis and lipogenesis in adipose tissue and the liver. m^6^A RNA methylation is partially involved in the regulation of fat metabolism in diet supplemented with excessive but not deficient BCAA.

## Data Availability

All data used in the current study are available from the corresponding author on reasonable request.

## Abbreviations

AAs: Amino acids
BCAA: Branched-chain amino acids
CP: Crude protein
H-BCAA: High dose BCAA
HDL-C: High-density lipoprotein-cholesterol
HNRNP: Heterogeneous nuclear ribonucleoprotein proteins
L-BCAA: Low BCAA dose diet
LDL-C: Low-density lipoprotein-cholesterol
LP: Low-protein
m^6^A: N6-methyl-adenosine
N-BCAA: Normal dose BCAA diet
NRC: National Research Council
RT-PCR: Real-time polymerase chain reaction
SID: Standardized ileal digestible
TC: Total cholesterol
